# Predictors of participation in risk-based prostate cancer screening

**DOI:** 10.1371/journal.pone.0200409

**Published:** 2018-07-10

**Authors:** Marie Koitsalu, Martin Eklund, Jan Adolfsson, Mirjam A. G. Sprangers, Henrik Grönberg, Yvonne Brandberg

**Affiliations:** 1 Department of Oncology-Pathology, Karolinska Institutet, Stockholm, Sweden; 2 Department of Medical Epidemiology and Biostatistics, Karolinska Institutet, Stockholm, Sweden; 3 Department of Clinical Science, Intervention and Technology (CLINTEC), Karolinska Institutet, Stockholm, Sweden; 4 Department of Medical Psychology, Academic Medical Center, University of Amsterdam, Amsterdam, The Netherlands; University of Zurich, SWITZERLAND

## Abstract

**Background:**

Implementation of risk-based prostate cancer screening has been proposed as a means to reduce the harms of PSA screening. Little is known, however, about the factors influencing men’s decision to attend a prostate cancer screening based on a risk assessment.

**Method:**

We sent postal invitations with a login to a survey to 10.000 men, three months before invitation to a risk-based prostate cancer screening. Prostate cancer specific worry, prostate cancer-related knowledge, health behaviour, and health related quality of life were used as predictors of subsequent participation. Participation to risk-based prostate cancer screening was defined as providing a blood sample for the STHLM3 trial, a study evaluating a risk-based model that predicts the risk for aggressive prostate cancer.

**Results:**

With a response rate of 20%, 1.347 men (70%) participated in ensuing risk-based prostate cancer screening three months later whereas 568 men (30%) declined participation in the STHLM3-study. These decliners reported less worry and feeling less vulnerable to prostate cancer and responded “Do not know” more often than participants when asked questions about prostate cancer knowledge. Participants reported greater benefits of prostate testing (p = 0.0005), less barriers to prostate testing (p<0.0001), and higher intention to attend prostate cancer testing (p<0.0001) than decliners. Finally, participants reported better overall health than decliners (p<0.0001).

**Conclusion:**

Prostate cancer worry, PC knowledge, health behaviour and quality of life were identified as predictors of participation in risk-based prostate cancer screening. Targeting these predictors may improve the participation rates. These results can inform policymaking for future population-based prostate cancer screening programs that should address potential worry in men and lack of knowledge about prostate cancer.

## Introduction

Prostate cancer (PC) is the second most common cancer in men worldwide [1]. The introduction of mass-screening programs is controversial [2]. The harms from testing with prostate specific antigen (PSA) in a screening setting have been judged to outweigh the benefits [3, 4]. Thus, PSA-testing has not yet been adopted by any governmental body as a structured and organized population screening method [4]. Implementation of risk-based prostate cancer screening has been proposed as a mean to reduce the harms of PSA screening [5]. By stratifying by PC risk, screening frequency can be determined, and individuals at highest risk of developing PC, and thus candidate for biopsy, identified. Little is, however, known about the factors influencing men’s decision to attend risk-based prostate cancer screening (PCS). Better understanding of predictors of participation to risk-based PCS is needed to assist in planning for future population based PCS in order to optimize attendance.

The aim of this paper is to identify relevant predictors (PC worry, knowledge about PC, health behaviour, and health related quality of life (HRQoL)) of participation in risk-based PCS.

## Materials and methods

### Study design

The study was embedded in the STHLM3-study, a population-based diagnostic study of almost 150.000 men aged 50–69 years, investigating whether a panel of biomarkers would more effectively identify men with PC compared with testing with PSA [6]. Participants for STHLM3 were randomly selected by date of birth from the Swedish Population Register kept by the Swedish Tax Agency. Men, who choose to participate in the STHLM3-study, visited one of the 67 laboratories in Stockholm collaborating with STHLM3 in order to provide blood samples for the PC risk assessment. The STHLM3 model uses a combination of plasma protein biomarkers, genetic polymorphisms, and clinical variables. The participants received a response letter based on the test results. The letter informed about the test by providing one of the following three recommendations: (1) Low risk with the recommendation to perform a new test in ten years; (2) A normal risk with the recommendation to have a new test in 2 years or (3) An increased risk of prostate cancer with the recommendation to consult an urologist for further examination and prostate biopsy. The results of the STHLM3 trial showed that the STHLM3 model performed significantly better than PSA alone for detection of cancers with a Gleason score of at least 7, and fewer men needed to undergo unnecessary biopsies [6].

The present study employed a prospective design. In January 2014, invitations to complete a web-survey were sent to 10.000 men who were due to be invited to participate in STHLM3 during the month of April 2014. The invitation letters were sent by mail and contained information about the present study and a login to the web-survey consisting of four questionnaires described below. No reminders were sent and no incentives were given. Respondents who replied ‘Yes’ or ‘Do not know’, when asked if previously diagnosed with PC, were excluded from this study. Information on subsequent participation was obtained from the STHLM3 database.

### Measures

Participation in a risk-based PCS, the outcome variable, was defined as providing blood for PC-testing within the STHLM3-study. To ensure applicability to the target population, we selected existing items from standardized questionnaires for the predictor variables. The web-survey covered four main areas:

#### 1. Prostate cancer-specific worry and perceived vulnerability

Worry about PC was measured by two items adapted from Watson et al. [7], and an additional item about the extent to which participants’ daily life is impacted by PC worry. Three items measured men’s perception of the risk of developing PC, i.e. their perceived vulnerability. Two were adapted from Steginga et al. [8], and one from Katz et al. [9]. The questions and response options are found in Table 1.

**Table 1:** Men’s worry and perceived vulnerability to prostate cancer (PC) by participation to risk-based PC screening, three months before invitation to screening.

	Participants		Decliners		P-value
	N = 1347	%	N = 568	%	
*Worry scale*					
How much do you worry about PC?					
Not at all	199	15	114	20	
A little	914	68	355	63	
A lot	215	16	85	15	
Very much	17	1	10	2	0.02 ^a^
Do not know	2	> 1	4	> 1	0.01 ^b^
How much of a problem is PC worry?					
Not at all	669	50	301	53	
A little	564	42	219	39	
A lot	99	7	42	7	
Very much	9	> 1	3	> 1	0.6 ^a^
Do not know	6	> 1	3	> 1	0.7 ^b^
How much is your daily life affected by PC worry?					
Not at all	1058	79	445	78	
A little	252	19	98	17	
A lot	26	2	18	3	
Very much	3	> 1	5	> 1	0.07 ^a^
Do not know	8	> 1	2	> 1	0.1 ^b^
*Perceived vulnerability*					
What do you think is your risk of getting PC?					
None	10	1	10	2	
Small risk	408	30	192	34	
Moderate risk	676	50	253	45	
High risk	102	8	49	9	
Very high risk	11	> 1	9	2	0.03 ^a^
Do not know	140	10	55	10	0.05 ^b^
How likely do you think it is that you will develop PC in the next 5 years?					
Very low	170	13	100	18	
Somewhat	720	53	297	52	
Moderate	198	15	61	11	
Very high	4	> 1	6	1	0.001 ^a^
Do not know	255	19	104	18	0.003 ^b^
In comparison to other men of your age and background, do you think you are more or less likely to get PC?					
Much less	23	2	19	3	
Less	205	15	91	16	
About the same	855	63	341	60	
More	106	8	48	8	
Much more	7	> 1	5	> 1	0.1 ^a^
Do not know	151	11	64	11	0.2 ^b^

^a^ Fisher’s exact test performed excluding the men answering “Do not know”.

^b^ Fisher’s exact test performed including the men who answered “Do not know”.

#### 2. Knowledge about prostate cancer

PC knowledge was measured by using the six-item questionnaire designed for men without a history of prostate cancer used by McNaugthon-Collins et al. [10]. The questions and response options are presented in Table 2.

**Table 2:** Prostate cancer (PC) knowledge by participation to risk-based PC screening, three months before invitation to screening.

	Participants		Decliners		P-value
	N = 1347 ^a^	%	N = 568	%	
How many men with early-stage PC do you think will die of the disease?					
Most or all will	12	> 1	3	> 1	
About half	139	10	63	11	
Most will not ^†^	1084	81	432	76	0.6 ^b^
Do not know	112	8	70	12	0.04 ^c^
Does active treatment for early-stage PC extend life?					
Very sure it can	683	51	279	49	
Pretty sure it can ^†^	594	44	233	41	
Not sure	35	3	28	5	
Pretty sure it cannot	4	> 1	2	> 1	
Very sure it cannot	8	> 1	4	> 1	0.1 ^b^
Do not know	23	2	22	4	0.01 ^c^
How many men with elevated PSA levels do you think have PC?					
Most or all do	73	5	25	5	
About half	510	38	179	31	
Most do not ^†^	393	29	169	30	0.2 ^b^
Do not know	371	27	195	34	0.01 ^c^
Do you think an infection or inflammation of the prostate can elevate PSA levels?					
Yes ^†^	556	41	223	39	
No	132	10	42	8	0.3 ^b^
Do not know	658	49	303	53	0.1 ^c^
Do you think a large prostate can elevate PSA levels?					
Yes ^†^	672	50	267	47	
No	177	13	58	10	0.3 ^b^
Do not know	497	37	243	43	0.03 ^c^
Do you think a prostate biopsy can miss some cancer?					
Yes ^†^	575	43	241	43	
No	322	24	120	21	0.4 ^b^
Do not know	449	33	207	36	0.3 ^c^

PSA = prostate-specific antigen

^†^ Denotes correct answer.

^a^ Numbers for individual items vary slightly because of nonresponse.

^b^ Fisher’s exact test performed excluding the men answering “Do not know”.

^c^ Fisher’s exact test performed including the men who answered “Do not know”.

#### 3. Attitudes and health behaviour

Attitudes towards prostate cancer screening and health behaviour was measured by a questionnaire aiming at identifying predictors of attendance for PSA screening tests and prostate biopsy [11]. This 26-item questionnaire comprises six scales: Perceived threat of developing PC (2 items), Perceived benefits of prostate testing (8 items), Perceived barriers to prostate testing (10 items), Intentions to undergo prostate testing (1 item), External influences on prostate testing decision making (3 items), and aspirations concerning general health (2 items). Responses for all items ranged from 1 to 5 (strongly disagree, disagree, neither agree nor disagree, agree, strongly agree). An English version of the questionnaire used for Attitudes and health behaviour can be found in the supporting information (S1 File).

#### 4. Health-related quality of life

The European Organization for Research and Treatment of Cancer (EORTC) Quality of Life Questionnaire (QLQ-C30) [12, 13] was used, which incorporates nine multi-items scales: five functional scales (physical, role, cognitive, emotional, and social); three symptom scales (fatigue, pain, and nausea and vomiting); and a global health and quality-of-life scale. Five single-item symptom measures are also included. Each item is scored from 1, “Not at all”; 2 “A little”; 3, “Quite a bit”; and 4, “Very much”, with the exception of items in the global quality-of-life scale, which range from 1 (“Very poor”) to 7 (“Excellent”). All EORTC scales were linearly transformed ranging from 0 to 100. The nausea and vomiting symptom scale as well as none of the single-items are not reported in the results, as they are not pertinent to this study.

All questionnaires were translated into Swedish by a certified translator and adapted to a web-based format. All original instruments have been used in previous international PC testing studies [8–11, 14].

#### Ethical approval

Ethical approval for this study was obtained from the Regional Ethical Review Board in Stockholm (Dnr 2012/572-31/1). As stipulated in the invitation letter, submission of the survey was interpreted as informed consent to participate.

#### Statistical analyses

For the “Attitudes and health behaviour” questionnaire, we added the possibility for participants to respond ‘Do not know’. Items in each scale were summed only if half or more of the responses in the scale were not composed of the response item “Do not know”. Summary scores were produced for each scale. As opposed to the original questionnaire [11], we used all items for all participants, and none were specific to subgroups.

Descriptive statistics were used to present the study sample. Differences for ordinal categorical data items were analysed using Fisher’s exact test. For analysis of two population means, independent Student t-tests were performed. The tests were two-sided and the level of significance was set to 0.05.

## Results

A total of 1.980 men (20%) responded to the questionnaires, three months before invitation to participate in a risk-based PCS. Of them, 65 men stated having previously been diagnosed with PC and were excluded from the study. A total of 1.347 men (70%) were categorized as *Participants*, i.e. provided blood for risk-based PC screening three months later, and 568 (30%) as *Decliners*. As only name and address were provided from the registry, no data on personal characteristics were available precluding non-respondent analyses.

### Prostate cancer-specific worry and perceived vulnerability (Table 1)

Three months before invitation to a risk-based PCS, a statistically significant difference was found between participants and decliners with respect to worry about PC. One out of five (20%) decliners stated not worrying at all, as opposed to 15% of the participants; whereas 63% of decliners worried ‘A little’, as opposed to 68% of the participants (p = 0.01). No between groups differences were found for “problems with PC-worry” or their daily lives being affected by PC-worry.

Participants were more likely than decliners to report perceiving a higher risk of developing PC (p≤0.05) and reported a higher likelihood of developing PC in the next five years (p≤0.003). There were no differences between participants and decliners with respect to their self-perceived risk in comparison to that of other men of the same age and background.

### Knowledge about prostate cancer (Table 2)

When excluding the response category “Do not know”, no statistically significant differences in knowledge were found between participants and decliners. After including the response item “Do not know”, statistically significant differences were found for four out of six questions. A larger proportion of decliners responded, “Do not know”. The levels of knowledge were generally low in both groups since ≤50% responded correctly to five of the six items. The exception was the question “How many men with early-stage PC do you think will die of the disease?” where ≥76% responded correctly.

### Health behaviour scale scores (Table 3)

**Table 3:** Health behaviour scale scores by participation to risk-based PC screening, three months before invitation to screening.

	Mean (SD) scale score		P-value ^c^
	Participants (n = 1271–1343) ^a^	Decliners (n = 525–563) ^a^	
A. Threats	6.66 (1.95)	6.49 (1.95)	0.10
B. Benefits	34.7 (5.51)	33.7 (5.98)	0.0005
C. Barriers	18.5 (5.66)	20.0 (6.93)	> 0.0001
D. Intention ^b^	1.33 (0.77)	1.61 (1.04)	> 0.0001
E. External influences	9.39 (3.58)	9.26 (3.61)	0.5
F. General health	7.94 (1.74)	7.74 (1.94)	0.03

SD: standard deviation; PC: prostate cancer

^a^ Expressed in ranges because participants who had responded ‘Do not know’ to more than half of the response items for a specific scale were excluded.

^b^ Low levels represents high levels of intention to attend PC testing.

^c^ t-test

No between group differences were found for two of the health behaviour scales (A. “Perceived threat of developing prostate cancer” and E. “External influences”). Participants indicated that they perceived larger benefits of PC testing (p = 0.0005), lower barriers to PC testing (p<0.0001), and had a higher desire for better general health (p = 0.03) than decliners. Moreover, participants reported a higher intention to participate in a PCS (p<0.0001).

### Health related quality of life subscales (Table 4)

**Table 4:** QLQ-C30 scale scores by participation to risk-based PC screening, three months before invitation to screening.

	Mean (SD) scale score		Diff (95% CI) ^a^	P-value ^b^
	Participants (n = 1347)	Decliners (n = 568)		
Global health status ^c^	81 (18)	77 (20)	-4 (-6 to -2)	> 0.0001
Physical functioning ^c^	97 (10)	96 (11)	-1 (-2 to 0)	0.2
Role functioning ^c^	94 (16)	93 (18)	-1 (-3 to 1)	0.3
Emotional functioning ^c^	88 (17)	85 (20)	-3 (-5 to -2)	0.0002
Cognitive functioning ^c^	90 (15)	89 (16)	-1 (-3 to 0)	0.10
Social functioning ^c^	94 (16)	92 (19)	-2 (-4 to 0)	0.02
Pain ^d^	11 (20)	12 (19)	1 (-1 to 3)	0.5
Fatigue ^d^	14 (18)	17 (21)	3 (2 to 5)	0.0002

SD: standard deviation: PC: prostate cancer

^a^ Mean difference (Part. vs. Decl.) and 95% confidence interval

^b^ t-test

^c^ High levels represents high levels of functioning and quality of life

^d^ High levels represents high levels of problems

Participants scored statistically significantly higher than decliners on “Global health status” (p<0.0001), “Emotional functioning” (p = 0.0002), “Social functioning” (p = 0.02), and lower on “Fatigue” (p = 0.0002). No statistically significant differences were found for the other functional subscales.

## Discussion

The present study identified predictors of risk-based prostate cancer testing by using a web-based questionnaire sent to men three months before invitation to STHLM3, a prostate cancer testing trial. The web-based survey was based on a set of questionnaires used previously in international PCS studies [8–11]. The men who later participated in STHLM3 and agreed to undergo PC testing, appeared to report more worry about PC, higher perceived risk of PC, higher levels of HRQoL, and higher intentions to participate in PCS than those who declined participation Perception of barriers and benefits of PCS also differed between the groups The men in both groups had comparable and low levels of knowledge about PC.

A slightly higher proportion of decliners reported not worrying at all and perceived their risk of developing PC as slightly lower than participants. The lack of information on socio-demographic, medical and family history of cancer in our study makes it difficult to interpret this difference. Similar results have, however, been found in previous studies. When comparing the two first questions concerning cancer worry with the results from Watson et al. [7] using the same questions, the level of worry was similar in both studies, but the quantification of how much of a problem cancer worry was differed. A higher proportion of men in our sample found cancer worry less of a problem. This difference between the studies is probably due to differences between the samples. In that study, women with a family history of breast cancer were included, whereas we had a population-based sample of men, aged 50 to 69. In addition, a study also measuring the perceived 5-year risk of PC (question n°5 in our worry questionnaire) showed similar results as our study concerning perceived risk, with a vast majority rating their perceived 5-year risk as low [9]. One study found that one of the major reasons for accepting PSA testing was men’s perception of low risk of prostate cancer [15]. Men not accepting PSA testing stated the same reason in that study. Whether worry is a reason to participate or abstain from participation in cancer screening remains to be further studied.

Previous studies have shown that prostate cancer knowledge is a predictor of participation in prostate cancer screening [14, 16, 17]. In our study, decliners responded ‘Do not know’ to a higher extent than participants. Further research is needed to understand to what extent socio-demographic and/or psychological variables explain those differences. However, the distribution of the responses as well as the high proportion of men responding “Do not know”, is in line with the results found by McNaughton et al. [10]. In addition, the knowledge level for participants and decliners reflected an overall lack of knowledge, which is in line with a number of studies that have demonstrated that men lack knowledge about the potential limitations and risks of PSA and PCS [10, 18–20]. Since the decision to undergo a test for PC relies largely on an educated decision and requires informed consent, more education and information before undergoing testing is highly needed. This should be considered when implementation of PCS is decided upon.

The participants in the present study reported a higher level of intention, a perception of more health benefits, and a higher desire for better general health. This is in concordance with a study published by Avery et al. using the same questionnaire for attitudes and health behaviour [11]. In that study, PSA test attenders, as opposed to PSA test refusers, reported similar attitudes as our participants. This finding would imply that the health behaviour regulating ensuing individual actions for PSA testing are similar to a risk-based PCS. Health beliefs and attitudes, as well as health intentions are considered to determine and regulate individual actions. More research is needed to investigate whether including risk assessment in PCS induces different health behaviours.

When comparing our HRQoL results with age matched reference values from a large sample of the Swedish population [21], our study sample scored higher (by more than 5 points) on physical and role functioning but reported similar levels on the other scales. Participants reported higher global health status, higher emotional functioning, as well as lower levels of fatigue compared to decliners. Those differences were, however, not clinically significant, and were probably due to the large sample size. Nevertheless, the present findings concur with those of other studies that have used quality of life questionnaires. Neither in The Rotterdam trial [22] nor in the ProtecT trial [23] and nor in the Finnish arm of the European randomized screening trial (ERSPC) [24] was health-related quality of life associated with the decision to attend PSA testing.

Our sample of responders seemed more inclined to participate in PCS than men in the regular population. As many as 70% of the men who responded to our web-survey participated in the subsequent STHLM3 study, as opposed to STHLM3’s participation rate of approximately 40% [6]. Hence, the responding sample may not be representative of men in the Swedish population. Since our study was performed prior to the men being invited to STHLM3, one explanation might be that our respondents had an initial interest in responding to PC questions. We did not have any information about family history of PC, which is one factor that might trigger the interest of PCT. Another possible explanation is that the web-survey itself triggered an interest in PCT, and thus increased the number of men who participated to STHLM3. In case of the latter option, there is room for improvement to increase interest in PCS and thus enhance the participation rates. Another study embedded within STHLM3 [25] showed how the use of a pre-notification postcard i.e. an introductory postcard sent a couple weeks prior to the invitation itself to STHLM3, increased participation rates. More research is needed to show how PC information may influence ensuing participation in PCS.

To the best of our knowledge, this is one of the first studies to date examining predictors of participation to a cancer screening programme using a risk-based strategy. The strengths of the present study are that it is population-based, and that the questionnaires were used in and developed for previous PCS-studies. Another advantage is the prospective design, which means that differences in predictor variables between PCS participants and decliners cannot be attributed to the outcome variable. Moreover, the outcome variable is an objective variable. The study has, however, also some limitations, of which the low response rate, which may have induced selections bias, and the lack of socio-demographic information, are the largest two problems. Whereas this study can identify attitudinal differences between participants and decliners, it cannot explain them in relation to demographic and medical history characteristics. The response rate found is in line with other web-based surveys targeting general population [26–28] Generalizations from this study should be made with caution, due to the high risk of selection bias.

### Practice implications

Implementation of PCS in non-symptomatic men is controversial. The present study reveals factors that differentiate between those who participate in risk-based PCS and those who decline. The general lack of knowledge in both groups highlights the need to increase educational efforts to enable men to make an informed decision whether they would participate in PCS or not. Better understanding of predictors of participation to risk-based PCS will help inform development of future health policy strategies in population-based PCS programmes by knowing where more resources are needed in order to increase participation to PCS. In addition, the results show differences in knowledge and attitudes between participants and decliners, but do not add to the discussion about the role of public PCS. The Swedish Board of Health and Welfare recently decided not to implement public prostate cancer screening [29]. The debate is, however, intense and there are groups (patients’ organizations and many physicians) who vehemently argue for public PCS. We think that it is of great importance to highlight the need for public education about the pros and cons of PCS if it should be implemented.

## Conclusions

This study has explored the implication of PC worry and perceived vulnerability to PC, PC knowledge levels, health-related quality of life and health belief attitudes with men’s participation in a risk-based PC screening. The results of the study indicate that attitudes are important components of men’s participation in PCS. Less worry was observed among PCS decliners, and they responded “Do not know” to a higher extent than participants when asked questions about PC knowledge. Participants expressed a higher desire for better general health, a higher level of intention to participate in PCS, and perceived more health benefits than decliners. However, the lack of socio-demographic and medical information among the respondents in our study sample precluded us from drawing conclusions as to what could explain the attitudinal differences observed. Caution must be taken when interpreting the results, as the response rate was low.

## Supporting information

S1 File Scales and items for the attitudes and health behaviour questionnaire. Responses for all items range from 1 to 5 (strongly disagree, disagree, neither disagree nor agree, agree, strongly agree).
(PDF)
