# Supplementary material for: Predictors of participation in risk-based prostate cancer screening
Source: PLoS One. 2018 Jul 10;13(7):e0200409. doi: 10.1371/journal.pone.0200409 (PMC6039032; doi:10.1371/journal.pone.0200409)
Supplement: S1 File — Responses for all items range from 1 to 5 (strongly disagree, disagree, neither disagree nor agree, agree, strongly agree). (PDF) [file pone.0200409.s001.pdf]

S1 File

Scales and items for the Attitudes and Health behaviour questionnaire. Responses for all items range from 1 to 5 (strongly disagree, disagree, neither disagree nor agree, agree, strongly agree) (PC: prostate cancer)

A. Perceived threat of developing prostate cancer (PC) (2 items)
   There is a high possibility that I will get PC in my lifetime.
   The older I get, the more likely I am to get PC.
B. Perceived benefits of PC testing (8 items)
   Having a PC test would give me peace of mind.
   Having a PC test would mean I wouldn't worry as much about PC.
   Having a PC test would allow finding PC early.
   Having a PC test would mean fewer men would die from PC.
   Having a PC test would decrease my chance of dying from PC.
   I want to discover health problems early.
   I believe that PC testing can help protect my health.
   Treatment for PC is more successful the earlier it is detected.
C. Perceived barriers to PC testing (10 items)
   Having a PC test would be an easy thing for me to do.
   Having a PC test would make me worry about PC.
   Having a PC blood test is painful.
   Having a PC test would take too much time/be inconvenient.
   I am worried that a PC blood test will show that I have PC.
   PC testing is not needed if you do not have symptoms.
   I am afraid that I might need to have treatment if I have a PC test that shows I have PC.
   I am worried that a prostate biopsy will show that I have PC.
   Having a prostate biopsy would be embarrassing.
   Having a prostate biopsy would be painful.
D. Intentions to undergo PC testing (1 item)
   I do not intend to undergo a PC blood test.
E. External influences on PC testing decision making (3 items)
   Knowing someone with PC could influence my decision to have a PC test.
   Recent publicity about PC could influence my decision to have a PC test.
   Talking to my doctor could influence my decision to have a PC test.
F. General health (2 items)
   Maintaining good health is extremely important for me.
   I search for new information to improve my health.
